# Increased resting-state cerebellar-cortical connectivity in breast cancer survivors with cognitive complaints after chemotherapy

**DOI:** 10.1038/s41598-021-91447-1

**Published:** 2021-06-08

**Authors:** Hye Yoon Park, Hyeongrae Lee, Joohyuk Sohn, Suk Kyoon An, Kee Namkoong, Eun Lee

**Affiliations:** 1 Department of Psychiatry and Institute of Behavioral Science in Medicine, Yonsei University College of Medicine, 50-1 Yonsei-ro, Seodaemun-gu, 03722 Seoul, South Korea; 2 Department of Mental Health Research, National Center for Mental Health, Seoul, Republic of Korea; 3 Division of Medical Oncology, Department of Internal Medicine, Yonsei Cancer Center, Yonsei University College of Medicine, Seoul, Republic of Korea

**Keywords:** Breast cancer, Human behaviour

## Abstract

Cognitive complaints after chemotherapy are common in breast cancer patients, but the neural bases for these complaints remain unclear. This pilot study explored resting-state functional connectivity (FC) as a marker of subtle cognitive changes in breast cancer patients who experience cognitive complaints. Chemotherapy-treated (n = 20, at least 6 months off therapy) and untreated (n = 17, disease-control) female breast cancer patients with cognitive complaints and healthy controls (n = 20) were recruited. The FC of the right dorsolateral prefrontal cortex was calculated, and any correlations between this FC and neuropsychological assessments were determined. Chemotherapy-treated patients with cognitive complaints displayed increased FC between the right dorsolateral prefrontal cortex and both the contralateral cerebellar lobule VII and the cerebellar vermis XI, compared to the disease-control and healthy-control groups, despite unimpaired neuropsychological performance. The increased FC was negatively correlated with executive function and attention in breast cancer survivors with cognitive complaints. Our pilot study findings provide evidence that cerebellar-cortical FC changes may be a pathophysiological basis for chemotherapy-related cognitive complaints. In addition, the FC changes have the potential to reflect minor or compensated cognitive function impairment in breast cancer patients.

## Introduction

Subjective cognitive complaints constitute one of the most common side effects of chemotherapy and also one of the most disturbing side effects in everyday life^1^. Even when chemotherapy is not applied directly to the central nervous system (CNS), cognitive difficulties can be a side effect of systemic chemotherapy in non-CNS tumor patients^2^. These cognitive effects of adjuvant systemic chemotherapy have been studied primarily in patients with breast cancer^3^, which is the most frequently diagnosed cancer among women in developed countries^4^. In light of the high percentage (17–75%) of breast cancer survivors who have reported cognitive complaints^5,6^, understanding and managing post-treatment cognitive changes in these patients are valid concerns.

Chemotherapy-treated breast cancer patients experience cognitive problems eight times more frequently than non-chemotherapy patients^7,8^. Even though chemotherapy has been regarded as the source for post-treatment cognitive complaints^9^, mild or undetected changes on neuropsychological assessment in post-chemotherapy patients^10^ make it challenge to uncover declines in cognitive function. Inconclusive findings and associations between cognitive complaints and psychological symptoms, like depression and anxiety^11^, also complicate the assessment of specific effects of chemotherapy on brain function.

Neuropsychological assessments alone have not been considered sensitive enough to determine minor cognitive changes^12,13^, so neuroimaging techniques are being used to determine neural correlates of cognitive complaints in cancer survivors. In particular, resting-state functional magnetic resonance imaging (rs-fMRI) is an objective method to examine brain network changes in breast cancer survivors who have undergone chemotherapy^14^. Prior studies comparing chemotherapy treated breast cancer patients with healthy controls have shown alterations in the executive network^15^; disrupted regional networks in the frontal, temporal, and striatal areas^16^; changes in the functional connectivity (FC) of the anterior cingulate cortex^17^ or the anterior hippocampus^18^; and alterations in amplitude of low-frequency fluctuation^14^. Kesler et al.^19^ demonstrated that damage to the default-mode network could discriminate chemotherapy treated patients from non-chemotherapy treated patients and healthy controls. Although the vast majority of studies focused on cognitive impairment instead of cognitive complaints when recruiting chemotherapy-treated patients, Piccirillo et al.^20^ compared chemotherapy treated breast cancer patients who self-report cognitive impairment to patients who did not self-report cognitive impairment. Disrupted frontoparietal FC was found in subjects who suffer from cognitive complaints^20^, and this finding suggested there may be alterations in FC in patients who have cognitive complaints compared to patients without cognitive complaints. To further our understanding of the effects of chemotherapy on cognitive complaints, patients who self-report cognitive complaints should be recruited, and within this group, patients who were treated with chemotherapy should be compared to patients who were not treated with chemotherapy. The targeted investigation of patients with cognitive complaints in their daily lives may not only increase the understanding of compromised cognitive function but also provide insight into the cognitive complaints themselves.

In prior FC studies investigating cognitive functions in breast cancer patients after chemotherapy, the dorsolateral prefrontal cortex (DLPFC) was investigated because of its well-known role in mediating a variety of cognitive processes^21^. During executive function tasks, reduced DLPFC activation was found in the chemotherapy treated patients compared to a non-chemotherapy treated patients^22^. Based on rs-fMRI results comparing breast cancer patients treated with chemotherapy to healthy controls, increased FC from the right DLPFC to the right middle temporal gyrus and the precuneus, as well as decreased FC of the right DLPFC with right inferior frontal gyrus, was demonstrated^15^. In a previous study which showed a separation of the DLPFC seed voxels^23^, the right posterior-dorsal subregion was strongly associated with working memory and cognitive action control. Therefore, by choosing the right posterior DLPFC (MNI coordinates^23^: x = 37, y = 33, z = 32, and radius = 10 mm) as the seed region to calculate its FC with the whole brain, the present pilot study explored using resting-state FC from the DLPFC as a marker of subtle or compensated cognitive changes in breast cancer patients who experience cognitive complaints. We hypothesized that cognitive complaints in chemotherapy-treated patients, which could not be detected using neuropsychological performance assessments, could be uncovered by assessing FC with rs-fMRI. We also expected that psychological factors, such as depression and anxiety, may partially account for cognitive difficulties, but that FC related to cognitive complaints would not be attributable to psychological factors.

## Materials and methods

### Participants

Breast cancer patients with cognitive complaints from the Severance Hospital of the Yonsei University Health System in Seoul, where they were treated with a standard-dose chemotherapy regimen (docetaxel/adriamycin/cyclophosphamide), were recruited by posting advertisements that we were seeking “female breast cancer patients (30–85 years old) who feel a decline in their cognitive functions” for a clinical study. Recruitment was also done via a website posting in an online community of breast cancer patients. There was restricted access to complete medical records, including detailed chemotherapy regimens, for the seven patients who were recruited through this online community. All breast cancer patients had completed chemotherapy at least 6 months prior to enrollment in the study, excluding continued hormonal therapy. Participants were excluded if they had (a) uncontrolled comorbid medical conditions; (b) history of psychiatric illness; (c) evidence of other types of cancer or recurrence of breast cancer; or (d) history of brain metastasis, brain injury, or any other neurological illness. Healthy controls who did not have cognitive complaints were recruited via advertisements on local websites. All participants were assessed using the Structured Clinical Interview for Diagnostic and Statistical Manual of Mental Disorders, 4th edition (SCID-I), the Hamilton rating scale for depression (HRSD)^24^, and the Hamilton anxiety scale (HAS)^25^. The participants included 20 patients with breast cancer who were treated with chemotherapy (group C+), 17 age-matched patients with breast cancer who were not treated with chemotherapy (group C−), and 20 healthy controls (group HC). Of the 57 participants, one non-chemotherapy patient and one healthy control were not included in the fMRI analyses because of data loss due to an MRI scanner error. One healthy control was excluded due to excessive motion artifacts (> 2 mm translation or > 2° rotation on a frame-to-frame basis). Thus, 54 participants were included in the final study analyses. A detailed flow diagram for the study is shown in Supplementary Fig. S1.

This study was carried out in accordance with the Declaration of Helsinki and was approved by the institutional review board at Severance hospital, Seoul, Korea (4-2014-0235). All subjects provided written informed consent to participate in the study.

### Neuropsychological assessment

The most frequent cognitive problems reported by breast cancer survivors include changes in attention, processing speed, executive function, and memory^9^. Therefore, we evaluated neurocognitive function in all participants using a battery of neuropsychological tests that covered the aforementioned domains: (1) attention and concentration (WAIS Digit span^26^); (2) processing speed (WAIS Digit symbol^26^); (3) executive function [Raven’s standard progressive matrices (SPM)^27^]; and (4) memory [Rey-Kim auditory verbal learning test (RAVLT)^28^].

### Image acquisition

The rs-fMRI data were acquired using a 3.0 T MRI scanner (Ingenia CX; Philips, Erlangen) equipped with a 32-channel head coil. Data were obtained while participants rested in the scanner with their eyes open and fixated on a white cross at the center of a screen. A T2*-weighted gradient echo planar imaging (EPI) sequence was used: repetition time (TR) = 2000 ms, echo time (TE) = 30 ms, flip angle = 90°, 31 interleaved slices, slice thickness = 4 mm, matrix size = 80 × 80, field of view (FOV) = 220 × 220 mm. All participants were recorded for 5.5 min (165 volumes), and two initial dummy volumes were obtained to ensure magnetization stability. High-resolution structural T1-weighted images using a turbo field echo sequence were acquired using the following parameter settings: spin-echo, TR = 9.9 ms, TE = 4.6 ms, flip angle = 8°, 220 coronal slices, slice thickness = 1 mm, matrix size = 224 × 224, FOV = 224 × 224 mm.

### Image preprocessing

The rs-fMRI data were analyzed using AFNI software (version 20.1.02^29^). The first three volumes of the functional dataset were discarded for signal stabilization. To suppress local spikes in the EPI signals, we performed de-spiking for each voxel time-series. Rigid-body registration of EPI volume to a base EPI volume was conducted for head motion correction. Physiological noise (i.e., respiratory and cardiac artifacts) were corrected using Physiological Estimation by Temporal Independent Component Analysis (PESTICA, https://www.nitrc.org/projects/pestica/), which is a tool to distinguish physiological signals from rs-fMRI data and then remove the physiological noise^30^. Slice-timing correction was performed for all slices within a volume. Co-registration to the high-resolution structural T1 images was performed, and then spatial normalization was carried out by affine transformations. The affine transformation parameters were obtained from normalization of the T1 images into Montreal Neurological Institute (MNI) space using the MNI avg152T1 template. T1 images were corrected for nonuniformity and striped skull before normalization into MNI space. The normalized T1 images were segmented into grey matter (GM), white matter (WM), and cerebrospinal fluid (CSF) by segmentation tool of FSL software (http://fsl.fmrib.ox.ac.uk/)^31,32^, and then the segmented images were binarized to create binary masks. Large ventricle (LV) masks were manually selected from the CSF mask. Voxels of EPI volume were resampled with 2 × 2 × 2 mm^3^ isotropic voxels, using nearest-neighbor interpolation. A temporal bandpass filtering was applied at 0.009 < f < 0.08 Hz. Nuisance signals, which included six head motion correction parameters and non-neural sources of variance from the eroded WM and LV masks, were regressed out. Framewise displacement (FD) was calculated based on the sum of the absolute value of the derivative of the six motion parameters^33^ and outlier volumes at time-points with an FD > 0.3 mm. In addition, the one preceding and the one following the time-point were excluded. Spatial smoothing was performed using an isotropic Gaussian kernel of full width at half-maximum of 6 mm.

### Functional connectivity analysis

The FCs between the averaged blood-oxygen-level-dependent time course from the right posterior DLPFC (MNI coordinates^23^: x = 37, y = 33, z = 32, and radius = 10 mm) and the time courses from the whole brain were computed. The right posterior DLFPC, which was masked with the GM mask, was used as a seed for regions of interest (ROIs). The correlation coefficients were converted into z-values using Fisher’s r-to-z transformation to obtain the FC strengths.

Analysis of variance (ANOVA) and analysis of covariance (ANCOVA) using age, years of education, depression scores, and anxiety scores as covariates were performed to compare FC strengths among the C+, C−, and HC groups. Statistical significance was set to a voxel-wise threshold of *P* < 0.001 within a cluster extent threshold of 65 voxels, which corresponds to a corrected family-wise error of *P* < 0.05. Significance thresholding for group analyses was conducted using 3dClustSim, which was available in the AFNI software suite. The mean values of FC strength were extracted in regions showing significant differences in FC.

### Group and correlative analyses

A one-way ANOVA, with group-type as a fixed factor, was used to assess differences among groups for normally distributed continuous variables. Kruskal–Wallis tests were performed for nonparametric variables. To assess group performance differences for the neuropsychological tests, ‘years of education’ was included as a covariate. We applied square transformation to Raven’s SPM and RAVLT-recall, cube transformation to RAVLT-recognition, square root transformation to HRSD, and fourth root transformation to HAS with a normal distribution. All variables had skewness levels that were acceptable for statistical analysis (< 1.0) after transformation. Pearson’s partial correlations, with an adjustment for years of education, were used to analyze correlations between the following parameters: neurocognitive domains and depression and anxiety in breast cancer patients and neurocognitive functions and FC. Correlations between neurocognitive functions and FC were then further explored with an adjustment for years of education and scores of depression and anxiety.

## Results

### Demographic and clinical characteristics

Demographic and clinical characteristics of breast cancer patients and healthy controls are summarized in Table 1. Age and years of education were not significantly different among the C+, C−, and HC groups; however, compared to the HC group, the breast cancer groups (C+ and C−) had significantly higher depression and anxiety scores, despite the exclusion of major psychiatric illnesses, as determined by SCID-I assessment. The C+ group did not differ from the C− group with regard to depression and anxiety scores.

**Table 1:** Demographic and clinical characteristics of patients and controls.

	C+ (n = 20)	C− (n = 16)	HC (n = 18)	Statistical analysis			
				Value	*P*-value	Post-hoc	*P-*value^†^
Age, mean (SD), years	52.0 (5.9)	50.8 (10.7)	52.8 (7.7)	F = 0.28	0.758		
Education, mean (SD), years	13.9 (3.4)	15.5 (3.5)	13.6 (2.7)	F = 1.76	0.182		
**Breast cancer stage**							
Carcinoma in situ	0	2					
I	7	11					
II	11	3					
III	2	0					
Hormonal therapy, no. (%)	13 (65.0)	14 (87.5)					
Radiotherapy, no. (%)	14 (70.0)	11 (68.8)					
Depression HRSD, mean (SD)	5.2 (4.9)	3.6 (2.2)	1.9 (2.1)	F = 4.37	0.006	C+ vs. C−	1.000
						C+ vs. HC	0.006
						C− vs. HC	0.061
Anxiety HAS, mean (SD)	4.5 (3.5)	2.6 (1.3)	1.5 (1.8)	F = 7.22	< 0.001	C+ vs. C−	0.269
						C+ vs. HC	< 0.001
						C− vs. HC	0.053

^†^Corrected *P*-values are derived from post-hoc comparisons with Bonferroni correction.

*C*+ patients treated with chemotherapy, *C−*, patients not treated with chemotherapy, *HC* healthy controls, *HAS* Hamilton anxiety scale, *HRSD* Hamilton rating scale for depression.

### Neuropsychological assessments

There were no significant group differences using the neuropsychological tests (Table 2). In the 36 breast cancer patients with cognitive complaints, lower attention/concentration values were correlated with depression (*r* =  − 0.37, *P* = 0.030) and anxiety symptoms (*r* =  − 0.41, *P* = 0.013), and lower executive function values were also correlated with higher scores for anxiety (*r* =  − 0.37, *P* = 0.031).

**Table 2:** Summary of neuropsychological assessments.

Domain	Test	C+ (n = 20)	C− (n = 16)	HC (n = 18)	*P-*value
Attention/concentration	WAIS digit span	11.4 (2.3)	12.4 (2.0)	10.9 (2.6)	0.440
Processing speed	WAIS digit symbol	11.4 (3.9)	12.5 (2.9)	12.5 (2.7)	0.404
Executive function	SPM	38.4 (9.7)	44.6 (9.3)	39.2 (10.0)	0.517
Memory	RAVLT-recall	13.5 (3.3)	12.8 (2.9)	13.8 (1.9)	0.341
	RAVLT-recognition	13.7 (2.8)	13.3 (2.2)	14.7 (1.0)	0.158

Values are means (SD). Higher scores on WAIS Digit span, WAIS Digit symbol, SPM, and RAVLT indicate better performance.

*C*+ patients treated with chemotherapy, *C−* patients not treated with chemotherapy, *HC* healthy controls, *RAVLT* Rey-Kim auditory verbal learning test, *SPM* Raven’s standard progressive matrices, *WAIS* Wechsler adult intelligence scale.

### Functional connectivity analyses

**Figure 1:**
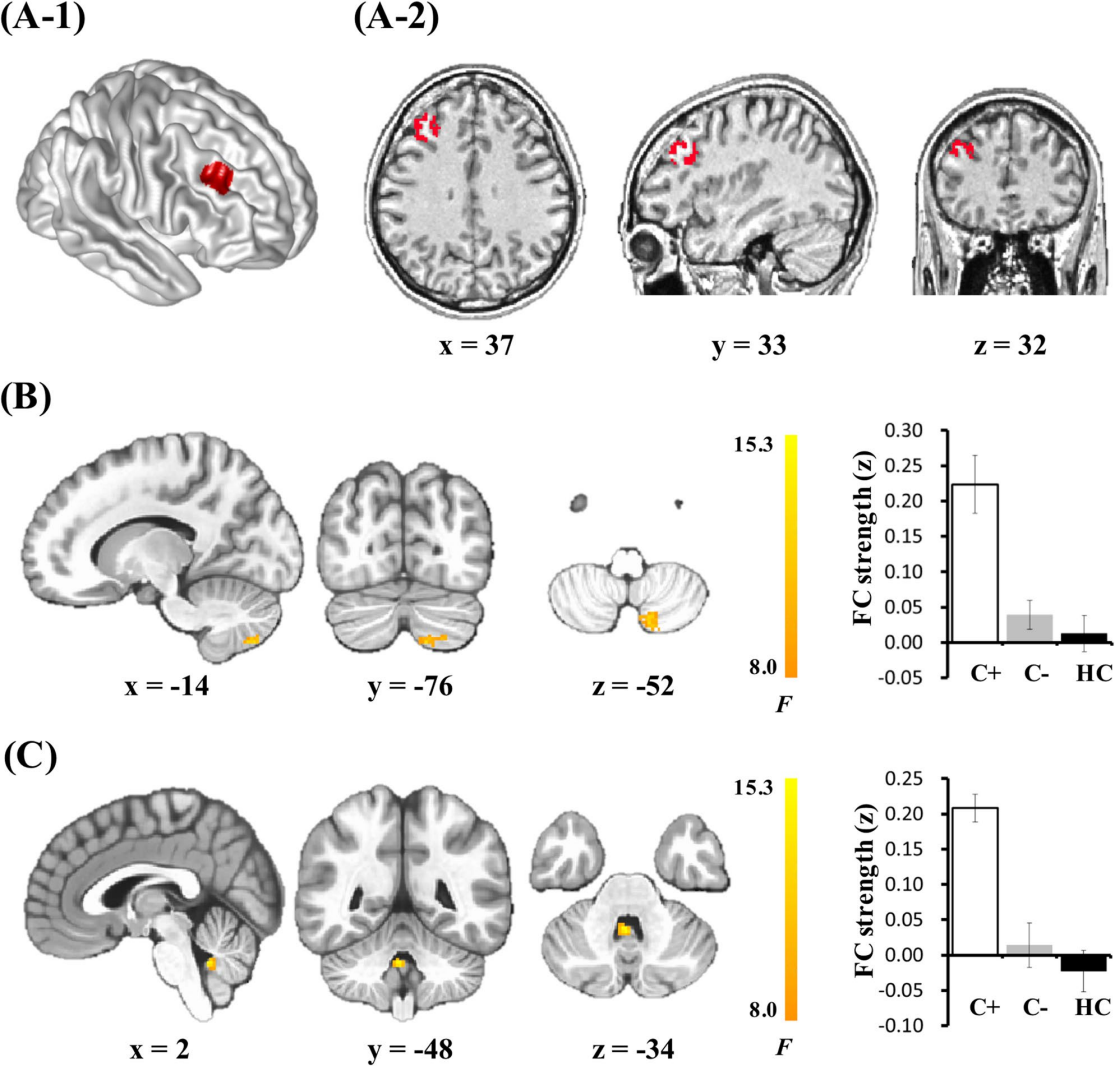
Group analysis results for FC of the right DLPFC. (**A**) The seed ROI in the right DLPFC (**A-1**) and its example on T1-weighted MRI of individual subject (**A-2**). The breast cancer patients treated with chemotherapy showed increased FC compared to control groups between the seed region of the right DLPFC and the left cerebellar lobule VII (**B**) and the cerebellar vermis XI (**C**). Brain maps of results of ANCOVA were superimposed on the MNI152 T1 template. The bar graph shows the average FC strength with the right DLPFC in the regions showing significant differences among groups. *FC* functional connectivity, *DLPFC* dorsolateral prefrontal cortex.

FC analyses using age, years of education, depression scores, and anxiety scores as covariates among the C+, C−, and HC groups demonstrated increased FCs between the right DLPFC seed region and the left cerebellar lobule VII (DLPFC-lobule VII) and between the DLPFC and the cerebellar vermis XI (DLPFC-vermis XI) in the C+ group compared to those in the control groups (C− and HC) (Fig. 1, Table 3). FC analyses without covariates were performed because the analyses with covariates had low statistical power due to small sample sizes. Both methods yielded similar results (Supplementary Table S1).

**Table 3:** Group analysis results for functional connectivity of the right DLPFC.

Brain area	Voxels	*F*	x	y	z
Cerebellar lobule VII, left	77	9.56	− 14	− 76	− 52
Cerebellar vermis XI	68	15.26	2	− 48	− 34

x, y, and z refer to left–right, anterior–posterior, and inferior-superior dimensions, respectively; *F* refers to the score at those coordinates.

*DLPFC* dorsolateral prefrontal cortex.

### Correlation analysis between neurocognitive performance and FC in breast cancer patients

**Figure 2:**
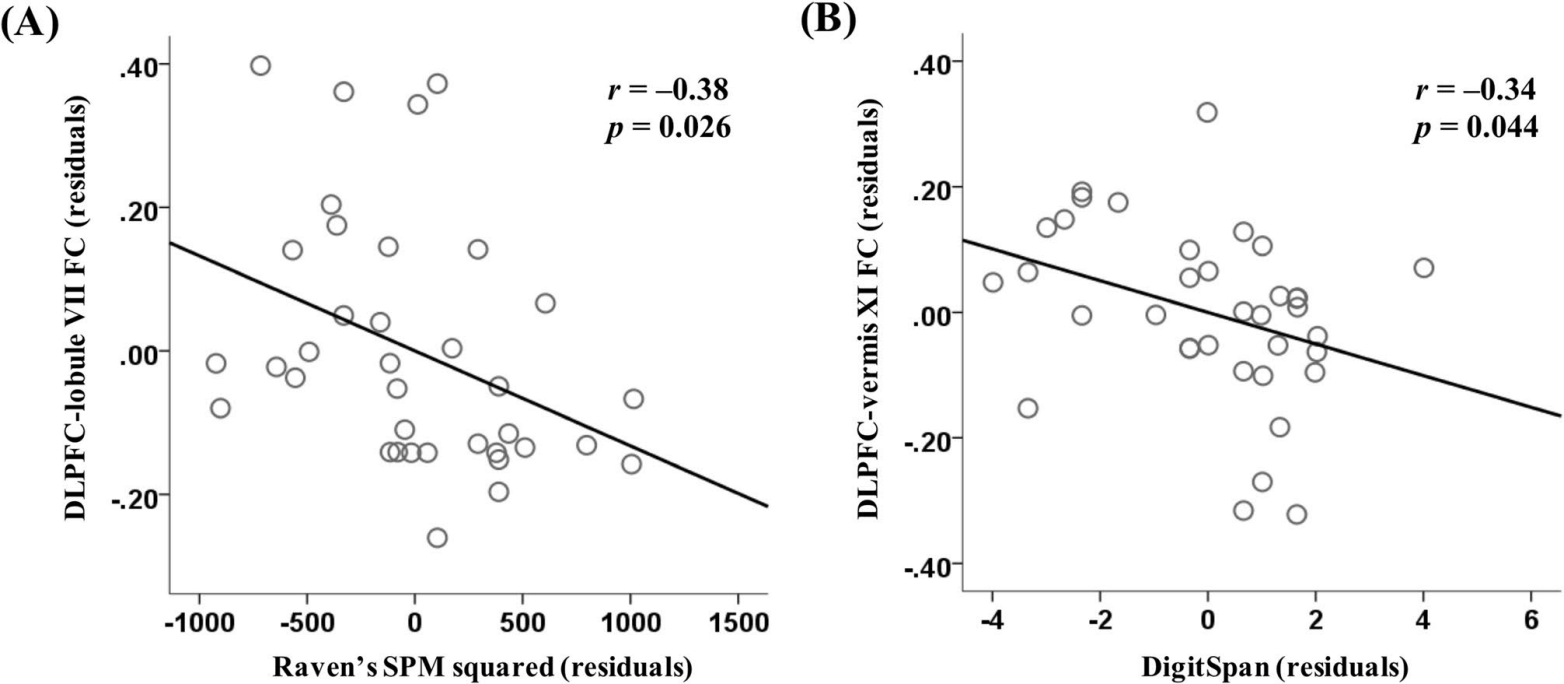
Correlation between neurocognitive performance and FC in breast cancer patients. (**A**) The significant partial correlation between the FC of the DLPFC-lobule VII and square-transformed scores of the Raven’s SPM, with an adjustment for years of education. (**B**) The significant partial correlation between the FC of the DLPFC-vermis XI and scores of the WAIS Digit span, with an adjustment for years of education. To visualize these partial correlations, variables were regressed onto years of education using a linear regression. Values reported in the scatterplot are non-standardized residuals. *FC* functional connectivity, *DLPFC* dorsolateral prefrontal cortex, *r* partial correlation coefficient, *SPM* standard progressive matrices, *WAIS* Wechsler adult intelligence scale.

In breast cancer patients with cognitive complaints (n = 36), the FC of the DLPFC-lobule VII was negatively correlated with executive function (*r* =  − 0.38, *P* = 0.026) (Fig. 2A). For FC of the DLPFC-vermis XI, attention/concentration was inversely correlated (*r* =  − 0.34, *P* = 0.044) (Fig. 2B). When the correlation analysis was adjusted for the scores of depression and anxiety, the correlation between FC of the DLPFC-lobule VII and executive function (*r* =  − 0.36, *P* = 0.039) remained.

## Discussion

This pilot study investigated resting-state FCs in the brains of breast cancer patients with cognitive complaints after chemotherapy compared to FCs in the brains of patients not treated with chemotherapy to reveal the effect of chemotherapy on cognitive complaints. The neurocognitive performance of breast cancer patients treated with chemotherapy was not significantly different from that of the disease-control and healthy control groups. However, a greater activation of the brain functional network area was shown in breast cancer survivors treated with chemotherapy. The FC analyses in our study used the scores of depression and anxiety as covariates, so the functional network changes could be considered unrelated to these psychological factors. We demonstrated an increased FC between the DLPFC and both the contralateral cerebellar lobule VII and the cerebellar vermis IX in the C+ group compared to both control groups. In addition, the FC strengths were correlated with attention and executive function. These results suggest that cerebellar-cortical FC changes in patients exposed to chemotherapy may be associated with their cognitive complaints.

Self-reported cognitive complaints are often overlooked or dismissed in clinical settings when they supported by objective testing results, as in our observation of non-significant differences in neuropsychological performance for breast cancer patients with cognitive complaints. By recruiting patients who self-report cognitive complaints and then comparing patients treated with chemotherapy to patients not treated with chemotherapy, the brain-network alterations seen in the chemotherapy-treated patients may suggest a potential biomarker for these complaints, which are related to the effects of chemotherapy, not to breast cancer or other cancer-treatments. Several candidate mechanisms have been proposed to explain such chemotherapy-associated brain changes. Increases in oxidative DNA damage, chemotherapy-induced hormonal changes, and the neurotoxic effects of inflammatory cytokines could all possibly increase the vulnerability of the brain to network changes associated with chemotherapy^34,35^. Consistent with recent studies showing widespread alterations in brain networks, rather than regionally specific effects of chemotherapy^16,36–38^, our FC-change findings offer additional convergent data supporting brain-network abnormalities as a mechanism for explaining cognitive complaints after chemotherapy.

Our findings showing an increased FC between the DLPFC and cerebellar regions in breast cancer survivors treated with chemotherapy may indicate alterations in cerebrocerebellar interconnections. Extensive cerebellar involvement in cognitive functions is well known^39,40^. Moreover, multiple cerebrocerebellar couplings have been observed involving the brain resting-state^41^. The cerebellar contributions to each cerebral network are distinct and selective^41^; for example, the neocerebellum is related to the executive control network (ECN), especially crus I and II with extensions into lobules VI–VII, and constitutes a crucial node for executive function^42^. Thus, the increased FC between DLPFC and lobule VII in our study may reflect a hyperactivation of the ECN due to compensatory mental efforts in chemotherapy-treated patients with cognitive complaints. The correlation between the FC of the DLPFC-lobule VII and executive function scores also suggests the possibility of compensatory neuroplasticity maintaining cognitive performance in chemotherapy-treated survivors. The involvement of the neocerebellum in emotion has also been documented^42^, however, controlling for depression and anxiety as covariates in our FC analysis suggested that these FC changes were unlikely to be due to any differences in psychological factors among groups. The correlation between FC of the DLPFC-lobule VII and executive function scores even after controlling for depression and anxiety suggests a cognitive role of FC, regardless of psychological factors.

Although a role for the vermal IX area has yet to be resolved, a stronger FC with the default-mode network (DMN) compared to the prefrontal cortices was demonstrated in a previous rs-fMRI study of healthy participants^43^. Therefore, increased FC of the DLPFC-vermis IX area in our patients could suggest a disruption of distinct cerebellar contributions to the intrinsic cerebral network. The aberrant coupling of cerebrocerebellar networks may interrupt subtle cognitive processes, resulting in cognitive complaints of the patients with chemotherapy. The correlation between the FC of the DLPFC-vermis IX area and attention scores in our patient group increases the likelihood of aberrant FC involvement in cognitive disturbances. Because the exploratory correlation analysis adjusted for depression and anxiety found a non-significant correlation between FC of the DLPFC-vermis IX and attention scores, these psychological factors may play a role in the relationship between FC and cognitive function, which could be revealed by future studies including mediation analyses and larger sample sizes. A troublesome confounder has been that psychological distress can undermine cognitive function in cancer patients who have an elevated risk for depression and anxiety^12^, and a relationship between the perception of cognitive complaints and psychological factors has also been documented^11,12^. In our study, high scores for depression and anxiety were correlated with both attention and executive functions in the breast cancer groups (C+ and C−) compared to the HC group. Those correlations suggest that psychological factors that underlie cognitive complaints should be addressed in survivors who report cognitive problems in their daily lives. Moreover, researchers should be aware of the confounding effects of psychological symptoms on studies of cognitive complaints, which was the reason we used depression and anxiety as covariates when performing FC analyses.

The present pilot study has several limitations. One is the limited heterogenous sample in terms of chemotherapy regimens and disease stages. For example, we were unable to evaluate whether any functional network alterations were related to individual chemotherapy regimens. Additionally, although a previous study^44^ showed non-significant effects of hormonal changes on cognitive function in breast cancer patients, we did not control for menopausal status, the effects of estrogen^45^, or hormone-replacement therapy^46,47^ on cognitive decline. The cross-sectional design of the present study also limited our understanding of any causal relationships between brain functional network changes and subjective cognitive problems. Therefore, prospective studies with large and homogeneous populations are needed to yield a definite conclusion. Lastly, our FC analysis included the comparison between C+ and C−, which was necessary to elucidate the effect of chemotherapy in patients with cognitive complaints; however, the effects of breast cancer or cognitive complaints themselves could not be determined. An elaborate 2 × 2 × 2 design (‘breast cancer patients vs. healthy individuals’ × ‘with vs. without cognitive complaint’ × ‘C+ vs. C−’) could provide a more thorough understanding by extensively controlling potential confounding effects.

In conclusion, we have demonstrated an increased resting-state FC between the DLPFC and cerebellar regions in breast cancer survivors who experienced cognitive complaints after chemotherapy treatment. Although many of these survivors still scored in the normal range on neuropsychological tests, the correlation between altered FC and cognitive performance suggests that cognitive complaints may be the result of compensatory processes of brain networks.

## Supplementary Information

Supplementary Information.
