# Supplementary material for: Increased resting-state cerebellar-cortical connectivity in breast cancer survivors with cognitive complaints after chemotherapy
Source: Sci Rep. 2021 Jun 8;11:12105. doi: 10.1038/s41598-021-91447-1 (PMC8187392; doi:10.1038/s41598-021-91447-1)

**Online Supplementary Material**

**Title:** Increased resting-state cerebellar-cortical connectivity in breast cancer survivors with cognitive complaints after chemotherapy

Running Title: Cognitive complaints after chemotherapy

**Authors:** Hye Yoon Park^a,+^, Hyeongrae Lee^b,+^, Joohyuk Sohn^c^, Suk Kyoon An^a^, Kee Namkoong^a^, Eun Lee^a,*^

Author Affiliations:

^a^Department of Psychiatry and Institute of Behavioral Science in Medicine, Yonsei University College of Medicine, Seoul, Republic of Korea

^b^Department of Mental Health Research, National Center for Mental Health, Seoul, Republic of Korea

^c^Division of Medical Oncology, Department of Internal Medicine, Yonsei Cancer Center, Yonsei University College of Medicine, Seoul, Republic of Korea

^+^These authors contributed equally to this study.

***Corresponding Author:**

Eun Lee, M.D., Ph.D.

Department of Psychiatry, Yonsei University College of Medicine

50-1 Yonsei-ro, Seodaemun-gu, 03722 Seoul, South Korea

Email: leeeun@yuhs.ac

Telephone: +82 2 2228 1622

Fax: +82 2 313 0891

**Supplementary Table 1.** Group analysis without covariates for functional connectivity of the right DLPFC

| **Brain Area** | **Voxels** | ***F*** | **x** | **y** | **z** |
| --- | --- | --- | --- | --- | --- |
| Cerebellar lobule VII, left | 73 | 12.66 | −24 | −78 | −50 |
| Cerebellar vermis XI | 74 | 14.94 | 10 | −56 | −28 |

**Supplementary Figure 1.** Study flow diagram.


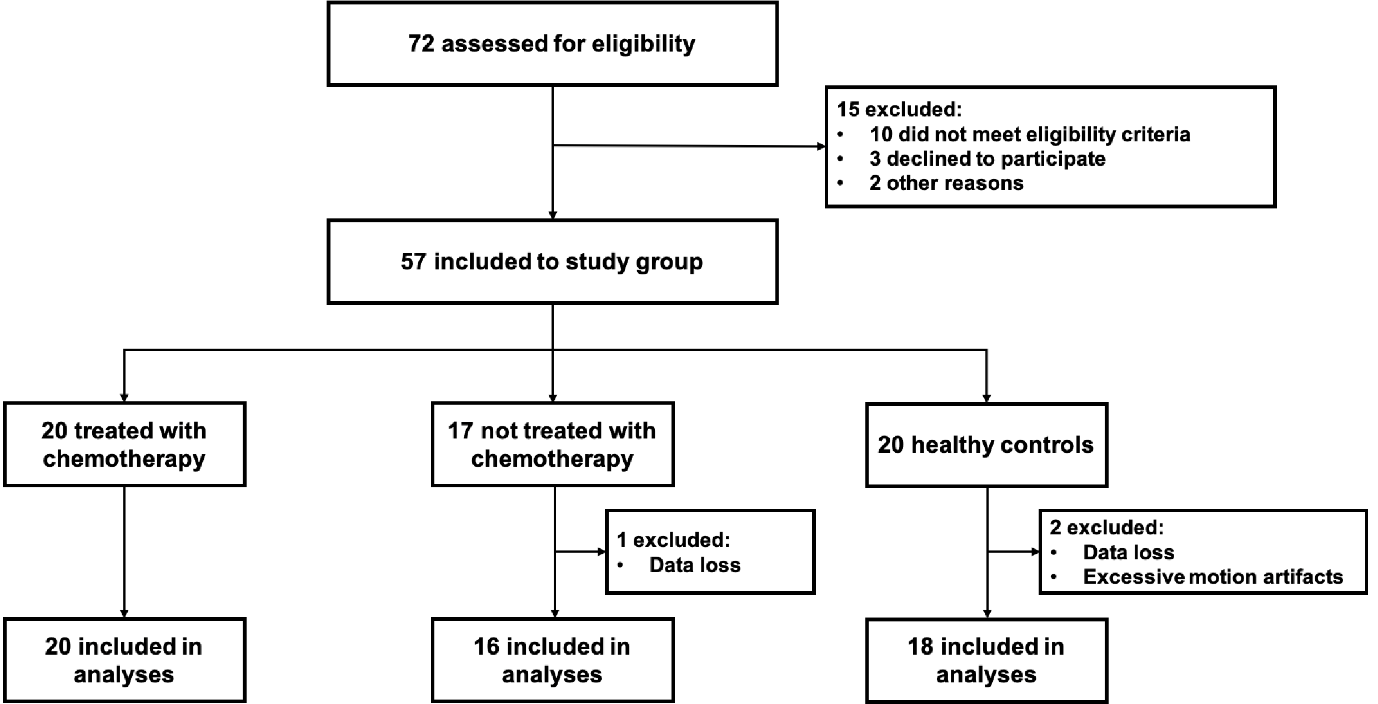

Supplement: Supplementary file 1 — Supplementary Information. [file 41598_2021_91447_MOESM1_ESM.docx]
